# 33886 Deregulation of the LANCL2 Pathway in Alzheimer's Disease

**DOI:** 10.1017/cts.2021.431

**Published:** 2021-03-30

**Authors:** Emily Birnbaumria, Maria Figueiredo-Pereira, Patrica Rockwell, Claire Henchcliffe, Peter Serrano

**Affiliations:** 1 CUNY Graduate Center, Hunter College, Weill Cornell Medicine; 2 Hunter College; 3 Weill Cornell Medicine, UC Irvine School of Medicine

## Abstract

ABSTRACT IMPACT: My work focuses on understanding the role of an immunomodulatory pathway in Alzheimer’s disease as a potential therapeutic target. OBJECTIVES/GOALS: My long-term goal is to develop effective drugs to halt the progression of Alzheimer’s disease (AD). The overall objective of this translational work is to study the deregulation of the Lanthionine Synthetase C-like 2 (LANCL2) immunomodulatory pathway in AD. I hypothesize that stimulation of the LANCL2 pathway will inhibit progression of AD. METHODS/STUDY POPULATION: These studies use a transgenic rat model of AD, and a LANCL2-binding drug, BT-11, that crosses the BBB. Tg-AD Fisher 344 rats express human APP with the Swedish mutation and human presenilin-1 with the ? exon 9 mutation. Tg-AD rats develop age-dependent AD-like pathology. Rodent chow containing BT-11 (10mg/Kg bw) was administered orally from 5 months of age (pre-pathology) to 11 months of age. Eight groups of rats (8 rats/group) were included: WT and Tg-AD x 2 sexes x 2 conditions (BT-11 treated and untreated). Rats are analyzed for: (1) Spatial learning and memory with an active place avoidance test. (2) AD pathology including amyloid plaques and activated microglia, with hippocampal immunohistochemistry. (3) LANCL2-signaling and other AD relevant pathways, with hippocampaI western blot and RNAseq analyses. RESULTS/ANTICIPATED RESULTS: I predict that BT-11 will prevent and/or mitigate some aspects of AD pathology displayed by the AD transgenic rats (Tg-AD). Tg-AD rats treated with BT-11 should perform better in the spatial learning and memory tasks, than the untreated Tg-AD rats. I also expect that the immunohistochemical analysis will reveal reduced pathological hallmarks, including amyloid plaques and reactive microglia, in hippocampal tissue of BT-11 treated versus untreated Tg-AD rats. Through western blot and RNAseq analyses, I will establish how BT-11 treatment affects the LANCL2 signaling cascade as well as other AD relevant pathways, in treated and untreated rats from both sexes and both genotypes. DISCUSSION/SIGNIFICANCE OF FINDINGS: My proposed translational research will address the potential of targeting the LANCL2 pathway to improve AD treatment. Discovering the effects of stimulating the LANCL2 pathway on AD pathology is a novel approach in AD drug development. I expect that my studies with BT-11 will positively influence therapeutic outcomes of this devastating condition.

